# Named entity recognition in aerospace based on multi-feature fusion transformer

**DOI:** 10.1038/s41598-023-50705-0

**Published:** 2024-01-08

**Authors:** Jing Chu, Yumeng Liu, Qi Yue, Zixuan Zheng, Xiaokai Han

**Affiliations:** 1 School of Automation, Xi’an University of Posts & Telecommunications, 618 West Chang’an Street, Chang’an District, Xi’an, 710121 China; 2 School of Astronautics, Northwestern Polytechnical University, 127 West Youyi Road, Beilin District, Xi’an, 710072 China

**Keywords:** Computer science, Aerospace engineering, Statistics

## Abstract

In recent years, along with the rapid development in the domain of artificial intelligence and aerospace, aerospace combined with artificial intelligence is the future trend. As an important basic tool for Natural Language Processing, Named Entity Recognition technology can help obtain key relevant knowledge from a large number of aerospace data. In this paper, we produced an aerospace domain entity recognition dataset containing 30 k sentences in Chinese and developed a named entity recognition model that is Multi-Feature Fusion Transformer (MFT), which combines features such as words and radicals to enhance the semantic information of the sentences. In our model, the double Feed-forward Neural Network is exploited as well to ensure MFT better performance. We use our aerospace dataset to train MFT. The experimental results show that MFT has great entity recognition performance, and the F_1_ score on aerospace dataset is 86.10%.

## Introduction

Unprecedented advances have been made in the domain of aerospace, with manned spaceflight technology being progressively commercialized, such as SpaceX's Dragon spacecraft. In combination with artificial intelligence, some of the complex operational steps in the aerospace domain will become simpler and autonomy can be exploited more in accordance with their prescribed tasks. Similar to humans, artificial intelligence first needs to learn prior knowledge. Text is one of the main storage forms of human knowledge. Therefore, it is particularly important to acquire knowledge accurately and quickly from a large number of aerospace text materials.

Named Entity Recognition (NER) is an essential technology to extract knowledge from documents. Its purpose is to extract words with actual meaning from text, including names of people, places, institutions and proper nouns. Unlike English, which has spaces as natural separators, Chinese entity recognition first needs to perform word segmentation on Chinese sentences, which makes Chinese named entity recognition more challenging. For example, if a sentence containing 12 characters is entity recognized, use *C* = {*c*_1_,*c*_2_,…,*c*_12_} to express. the result may be *w*_1_, *w*_2_ or *w*_3_ (*w*_3_ contains both *w*_1_ and *w*_2_), but only *w*_3_ is the correct result.

**Figure 1:**
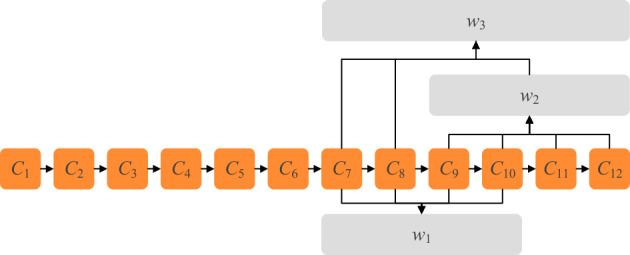
Use the lexicon to match the Chinese sentence to the words *w*_1_, *w*_2_ and *w*_3_, these Chinese words make it easier for the NER model to determine entity boundaries. Whether the entity in a sentence is *w*_1_ or *w*_2_ or *w*_3_ can be determined by the NER model using contextual semantics.

In order to reduce the impact of word segmentation errors, Zhang et al. proposed a Lattice structure that can consider both characters and words, and this structure was used on the Lattice-LSTM (Lattice Long-Short Term Memory)^1^. As shown in Fig. 1, the structure matches the sentence with the lexicon to obtain all potential words contained in the sentence, and then performs feature extraction for each character and the matched words in the sentence. The Lattice will use the contextual information to determine which of *w*_1_, *w*_2_ and *w*_3_ is the correct word segmentation result, avoiding the recognition failure caused by word segmentation errors. Li et al. modified the Lattice structure to be combined with Transformer-XL^2^, and proposed FLAT (Flat-Lattice-Transformer)^3^. FLAT uses a Flat-Lattice, which places the words matched from the lexicon at the end of the input sentence and determines the position of these words in the sentence through position encoding. However, this method ignores the radical information of Chinese characters.

As a character evolved from hieroglyphs, the radicals of Chinese characters usually contain a lot of information. This information could be used to further enhance the semantic information. Dong et al. used Bidirectional Long Short-Term Memory (Bi-LSTM) to decompose the Chinese character structure to obtain character-level embeddings and demonstrated the effectiveness of the method^4^. However, the Long Short Term Memory (LSTM) has insufficient parallel computing ability and its performance is lower than that of the Transformer.

To address these issues, we used a NER model based on Multi-Feature Fusion Transformer (MFT). The model is based on FLAT and uses a One-Dimensional Convolutional Neural Network (1D-CNN) to integrate information about the corresponding radicals of Chinese characters. The MFT uses features from Chinese characters, words and radicals to make it computationally efficient and reduce errors in word segmentation by introducing radical information to enhance semantic information. The structure of the Transformer has also been adapted to make it perform the task of named entity recognition better.

In order to recognize entities in aerospace contexts, the MFT model needs to be trained on the aerospace domain corpus. At present, there is no publicly available Chinese named entity recognition dataset in the aerospace domain. Therefore, within the scope permitted by law, we used the web crawler technology to obtain relevant data from Baidu Encyclopedia and other websites. To that end, we have produced an aerospace dataset that contains 30 K Chinese sentences to complete the model training and testing.

## Related work

Aerospace named entity recognition belongs to the specific field of named entity recognition, but it still belongs to the research in the field of named entity recognition. Deep learning has advanced rapidly in recent years and various named entity recognition methods based on deep learning have appeared. As one of the early deep learning models, LSTM was applied to the named entity recognition task by Hammerton^5^. However, LSTM only extracts features in a sentence from a single direction. To solve this problem, Huang et al. used BiLSTM that combined with Conditional random fields (CRF) for the entity recognition task and had achieved satisfactory results. In addition to temporal models that can be used for semantic modeling^6^, Collobert et al. used Convolutional Neural Networks (CNN) as NER model encoders to model local semantic features of sentences and generate corresponding labels with CRF as decoders^7^. Dos Santos used an improved CNN model for Natural Language Processing (NLP), 1D-CNN, to recognize entities. Experiments show that this improvement is very effective^8^. However, these methods only perform feature extraction on characters or words one by one. For this reason, Vaswani et al. proposed a Transformer model based on a self-attentive mechanism, which provides a new idea for named entity recognition. The method not only improves the recognition accuracy of the model, but also reduces the training time of the model^9^. Dai et al. believe that the modeling ability of long-term dependency is crucial to the language model, which is also the defect of Transformer, so they improved it and proposed Transformer_XL model, which improves the modeling ability of Long-Term dependency by 80%. However, Guo et al. believe that named entity recognition is different from other language models and should pay more attention to the modeling of local semantics^10^. They propose a lightweight Star Transformer model. Experiments show that this model is more suitable for NER tasks.

Chinese named entity recognition methods are classified into character-based named entity recognition methods and word-based entity recognition methods. Character-based approaches lose word information in sentences, and word-based approaches are more influenced by the quality of the segmentation. Liu et al. discuss character-based and word-based approaches separately and conclude that character-based approaches are empirically better choices^11^. However, some researchers have tried to combine the two methods by combining lexicon information on a character-based approach. Gui et al. proposed Lexicon Rethinking Convolutional Neural Network (LR-CNN), which uses a lexicon to assist the model in the determination of entity boundaries^12^. Zhang et al. proposed Lattice LSTM, which reinforces semantic and entity boundaries by using a lexicon. Gui et al. proposed a Lexicon-Based Graph Neural Network (LGN), where the graph neural network is used to introduce the latent word information matched by the dictionary into the model to complete the entity recognition task^13^. Li et al. proposed FLAT, which uses relative position encoding to recover lattice structure information. Since Lattice is compatible with Transformer, the performance of the model is further improved.

In terms of structural features of Chinese characters, Dong et al. introduced the structural information of Chinese characters into the NER model for the first time and used Bi-LSTM for the feature extraction of Chinese radicals; this method achieved the best performance on the MSRA dataset. Meng et al. used images of Chinese characters to assist in completing NER by leveraging the image information of Chinese characters to take advantage of the strokes and structural features of Chinese characters^14^.

There are also many named entity recognition works in the aerospace domain. Xu et al. crawled relevant texts from NASA's official website to produce a spacecraft named entity recognition dataset and used CRF to complete the entity recognition task^15^. Boan Tong et al. used the book World Spacecraft Encyclopedia as the data source for constructing the spacecraft-related dataset and performed migration learning through the Bert-BiGRU-CRF (Bidirection Gated Recurrent Unit, BiGRU) model to fine-tune the model parameters in the spacecraft domain corpus to accomplish the entity recognition task in the spacecraft domain^16^. Tikayat et al. developed an English-language aerospace dataset with which they fine-tuned BERT for better recognition performance in the aerospace domain^17^. In this paper, we will develop a Chinese aerospace dataset and propose a new recognition method based on the characteristics of Chinese.

## Aerospace dataset

**Figure 2:**
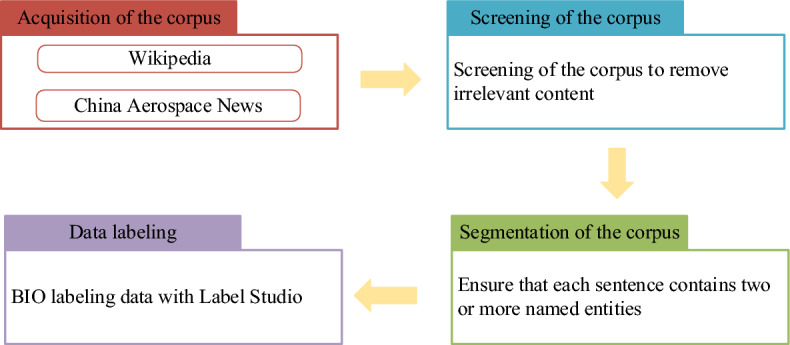
Extracting relevant corpus from Wikipedia and Chinese space news, screening and segmentation of the corpus and labeling it in BIO format using Label Studio.

Since there is no publicly available named entity recognition dataset in the aerospace domain, we use the crawler system to obtain relevant corpus from the data on Internet websites such as Wikipedia to the extent permitted by laws and use Label Studio for manual labelling. A dataset of aerospace domain with 29,953 sentences and 51,482 entities is made. The construction process of the aerospace dataset is shown in Fig. 2.

**Figure 3:**
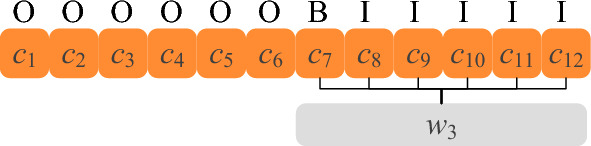
Annotation of sentences containing 12 Chinese characters using the BIO labeling method.

First, we use a crawler based on the Scrapy framework to obtain aerospace data from Wikipedia and China Aerospace News, then we filtered the corpus to remove contents that are not relevant to the domain. After that, we sliced the corpus in sentences and ensured that each sentence contained at least two aerospace entities. Finally, the corpus was labeled in the BIO format with the help of Label Studio. An example of the BIO Labeling format is shown in Fig. 3, where ‘B’ stands for ‘Begin’ and is used to annotate the head of the entity, ‘I’ stands for ‘Inside’ and is used to annotate the rest of the entity and ‘O’ is for ‘Outside’ and is used to annotate the non-entity.

Entities are categorized into aerospace companies and organizations (ACAO), Airports and spacecraft launch sites (AASLS), Type of aerospace vehicle (TOAV), Constellations and satellites (CAS), Space missions and projects (SMAP), Scientists and astronauts (SAA), aerospace technology and equipment (ATAE). 7 types. In this paper, 80% of the data in the dataset is used to train the model, 10% is used to validate the model, 10% is used to test the model. The main information of the aerospace dataset is shown in the Table 1.

**Table 1:** The main information of aerospace dataset.

Type	Train	Dev	Test
Sentence	24,000	2976	2977
Entity	41,222	5080	5180
ACAO	2023	254	206
AASLS	453	59	54
TOAV	3467	418	441
CAS	1413	165	153
SMAP	7380	897	930
SAA	550	55	84
ATAE	25,922	3232	3312

## Multi-feature fusion transformer

**Figure 4:**
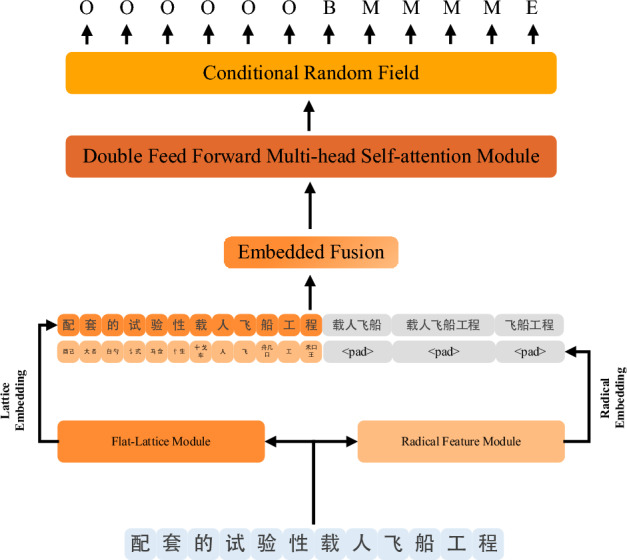
The flat lattice module and the radical feature module represent the embedding of the Chinese sentence respectively, and the double feed-forward multi-self-attention module encodes these embeddings, which are finally decoded by the conditional random fields to obtain the label sequence.

Since the word and radical information are very important features for Chinese characters. So in this paper we use MFT that can fuse these information as a named entity recognition model. The network structure of the MFT model is shown in Fig. 4. The model first extracts the radical embedding of Chinese characters through 1D-CNN, then fuses it with the Lattice sequence embedding output by the FLAT-Lattice model and encodes it as the input of the Flat-Lattice model, which is encoded as inputs to the Double Feed-forward Multi-head Self-attention (DFMS) encoder module, and finally decodes the corresponding label sequences by CRF. In the DFMS encoder module, MFT has exploited the structure of the Transformer by adding a Feed-Forward Neural Network (FFN) before the multi-headed self-attention module. This sandwich structure of the Transformer shows better performance in the NER.

### Flat-lattice module

**Figure 5:**
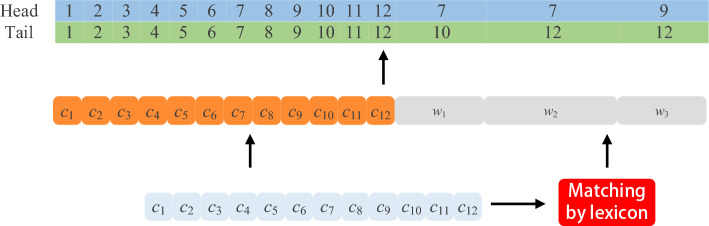
Flatten the lattice by using the head and tail positions of Chinese characters and words to record the position of each token in the lattice structure.

Similar to the Flat-Lattice module in the FLAT model, the Flat-Lattice module in the MFT uses a lexicon to match the input sentence to obtain the potential words contained in the sentence and encodes the positions of characters and these potential words in order to construct the Lattice. The structure of the Flat-Lattice module is shown in Fig. 5. For example, if a sentence containing 12 characters is entity recognized. Match the sentence with the lexicon to get the potential words *w*_1_, *w*_2_ and *w*_3_. These matched potential words are placed at the end of the sentence as candidates for the entities in the sentence, which together with the sentence form the lattice sequence *LS* = {*ls*_1_,…,*ls*_*n*_}. The tokens in the Flat-Lattice are then located using the head position and tail position to restore the Lattice structure information.

Next, for the Flat-Lattice sequence, we need to convert it to Flat-Lattice sequence embedding and encode it by positions. The Lattice sequence embedding *LE* = {*le*_1_,…,*le*_*n*_} can be obtained by matching *LS* in a pre-trained embedding table. Their positional embeddings, on the other hand, are calculated respectively by using Eqs. (1)–(7).

1 $$R_{ij} = {\text{ReLU}}((P_{{d_{ij} }}^{hh} \oplus P_{{d_{ij} }}^{ht} \oplus P_{{d_{ij} }}^{th} \oplus P_{{d_{ij} }}^{tt} )W_{p} )$$

2 $$P_{d}^{2k} = \sin (d/1000^{{2k/d_{emb} }} )$$

3 $$P_{d}^{2k + 1} = \cos (d/1000^{{2k/d_{emb} }} )$$

4 $$d_{ij}^{(hh)} = head[i] - head[j]$$

5 $$d_{ij}^{(ht)} = head[i] - tail[j]$$

6 $$d_{ij}^{(th)} = tail[i] - head[j]$$

7 $$d_{ij}^{(tt)} = tail[i] - tail[j]$$

where *R*_*ij*_ in Eq. (1) represents the relative position encoding between token* i* and token *j* with ⊕ representing the concatenation operator and *W*_*P*_ being the learnable parameter. ${P}_{{d}_{ij}}^{hh}$ represents the encoding of the relative distance between the head positions of token* i* and token *j*, ${P}_{{d}_{ij}}^{ht}$, ${P}_{{d}_{ij}}^{th}$ and ${P}_{{d}_{ij}}^{tt}$ have similar meaning, which is calculated using the same formula as the position code calculation in Transformer, ${P}_{{d}_{ij}}^{hh}$ as shown in Eqs. (2)–(3), where *k* represents the index of the position coding dimension, *d*_*emb*_ represents the position coding dimension, ${d}_{ij}^{hh}$ represents the distance between the head positions of token* i* and token *j*, ${d}_{ij}^{ht}$, ${d}_{ij}^{th}$, and ${d}_{ij}^{tt}$ have similar meaning, and they are calculated by Eqs. (4)–(7), where *head*[*i*] denotes the head position of token *i* and *tail*[*j*] denotes the tail position of token* j*.

### Radical feature module

In Chinese, some characters such as "river", "lake" and "sweat" are related to water, so they all contain the same radical. The radicals in Chinese characters are similar to the root affixes in English. As a kind of characters evolved from hieroglyphs, Chinese characters contain a lot of semantic features in their radicals. In order to use these semantic features to enhance the semantic information of sentences, Radical Feature Module splits each Chinese character into multiple radicals by radical dictionary and encodes these radicals by 1D-CNN to obtain the radical encoding of the corresponding Chinese character.

**Figure 6:**
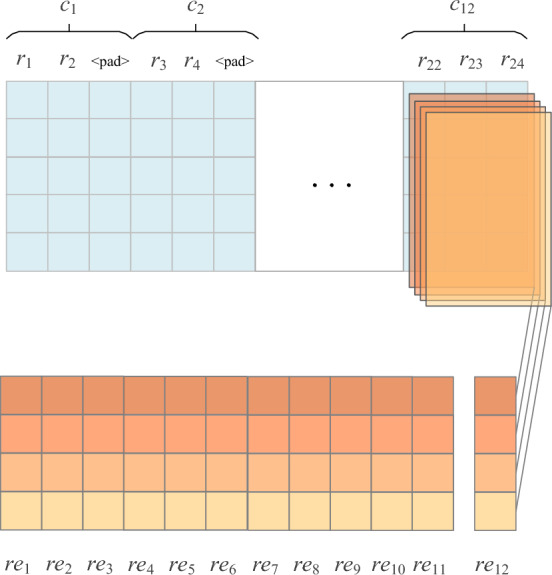
Extraction of the radicals in Chinese characters using 1D convolution to obtain the radical embedding for each character.

Take the radical encoding of a sentence containing 12 characters as an example. Each character in the sentence is matched in the radical dictionary to obtain the radical group corresponding to each character, where the character with the highest number of radicals contains 3 radicals, then the size of the convolution kernel of 1D-CNN is set to 3 and the step size is also 3. The remaining words with less than 3 radicals are filled in with "<PAD>", a symbol used exclusively for filling in deep learning. The convolution process is shown in Fig. 6. By this method, we can obtain the corresponding radical embedding sequence *RE* = {*re*_*1*_,…,*re*_*n*_} for the sentence.

Radical Feature Module only extracts the radical feature of the characters in the input sentence, and the radical feature of the potential words in the sentence is not extracted. This results in that the lengths of *LE* and *RE* are different. In order to facilitate the subsequent fusion of them, Radical Feature Module also uses "< PAD >" to fill in the radical sequence embedding *RE*, so that the lengths of *LE* and *RE* are consistent with each other.

Finally, the lattice sequence embedding and the radical feature sequence embedding are concatenated to obtain the sequence embedding *E* = {*e*_*1*_,…,*e*_*n*_}, as shown in Eqs. (8) and (9).

8 $$E = LE \oplus RE$$

9 $$E = LE + RE$$

### DFMS encoder module

**Figure 7:**
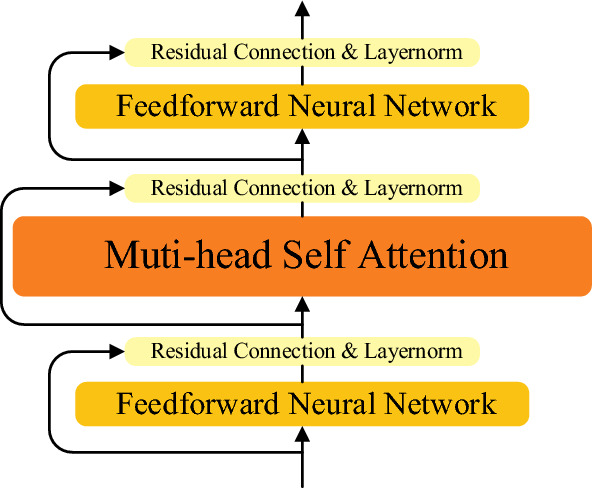
Using double feed-forward neural networks to clip multi-head self-attention modules with residual connections and layer normalization between them.

There are two kinds of neural networks in DFMS encoder, which are Self-Attention Neural Network and Feed-forward Neural Network. The structure is shown in Fig. 7, where the Self-attentive Neural Network is the same as the self-attentive network in Transformer_XL, which uses relative position coding, with the aim of improving the model's ability to model long-term dependencies. DFMS has a Double Feedforward Neural Network, with the self-attentive neural network added between them. This structure has proven to be effective in Conformer^18^. Residual connections and normalization are also required between each layer of neural networks.

We use the sequence embedding *E*, which fuses *LE* and *RE*, as the input to the DFMS encoder. As shown in Eqs. (10)–(14). Firstly, the sequence embedding *E* enters into the first layer of FFN calculation, and the output result enters into the Self-attentive Neural Network for self-attention coding after the layer normalization and residual connection. The coded result also needs the residual connection and layer normalization processing. In the end, after the second FFN calculation, the final encoding of the encoder is obtained.

10 $$E^{*} = E + (({\text{ReLU}}(EW_{1} + b_{1} ))W_{2} + b_{2} )$$

11 $$[Q,K,V] = E^{*} [W_{q} ,W_{k} ,W_{v} ]$$

12 $$S_{ij} = (Q_{i} + u)^{{\text{T}}} K_{j} + (Q_{i} + v)^{{\text{T}}} R_{ij} W_{R}$$

13 $$A = E^{*} + {\text{Softmax}}(S)V$$

14 $$X = A + (({\text{ReLU}}(AW_{3} + b_{3} ))W_{4} + b_{4} )$$

where *W*_*q*_, *W*_*k*_,* W*_*v*_ and *W*_*R*_ are the query mapping matrix, the key mapping matrix, the value mapping matrix and the position mapping matrix respectively, all of which are learnable parameters. With *W*_*q*_, *W*_*k*_ and* W*_*v*_, the sequence embedding *E* is mapped to the query matrix *Q*, the key matrix *K* and the value matrix *V* respectively. In Eq. (12) *u* and *v* are also learnable parameters that are used to ensure that the attentional bias of the query vector remains constant for different tokens^2^. *W*_1_, *W*_2_, *W*_3_, *W*_4_, *b*_1_, *b*_2_, *b*_3_ and *b*_4_ are learnable parameters of the Feedforward Neural Network.

### CRF decoder module

Conditional random fields are often used in machine learning-based named entity recognition methods. Benefiting from its excellent performance, CRF is usually used as decoders based on neural network named entity recognition models. CRF is a conditional probability distribution model that can be used to solve prediction problems. Cuong et al. propose that CRF can be used to solve the labeling problem and derive the most sensible label in conjunction with the semantic^19^. In the named entity recognition task, CRF takes the input sequence of observations as the set of random variables *X* and the output sequence of labels as *Y*. As shown in Eqs. (15)–(16), for a sequence *X* = {*x*_1_,…,*x*_n_}, the corresponding sequence of labels is *Y* = {*y*_1_,…,*y*_n_}. The probability of y is *P*.

15 $$P(y|x) = \frac{{\exp (\sum\limits_{i,k} {\lambda_{k} t_{k} (y_{i - 1} ,y_{i} ,x,i)} + \sum\limits_{i,l} {u_{l} s_{l} (y_{i} ,x,i)} )}}{Z(x)}$$

16 $$Z(x) = \sum\limits_{y} {\exp (\sum\limits_{i,k} {\lambda_{k} t_{k} (y_{i - 1} ,y_{i} ,x,i)} + \sum\limits_{i,l} {u_{l} s_{l} (y_{i} ,x,i)} )}$$

where *t*_*k*_ is the transfer eigen-function and *s*_*l*_ is the state eigenfunction, taking values of 1 or 0. *λ*_*k*_ and *u*_*l*_ are the corresponding weight coefficients, which are learnable parameters.

## Experiment

In the experiments of this paper, we compare the performance of the MFT model with some mainstream named entity recognition models on our aerospace dataset.

In addition, in order to verify whether the MFT model is only effective on our aerospace dataset, we also conduct performance comparison experiments on some commonly used and public named entity recognition datasets such as Weibo and Resume datasets. Finally, we conduct an effectiveness study on the MFT model to verify the effectiveness of our model structure.

### Evaluation indicator

Common evaluation criteria used in Named Entity Recognition tasks are precision (P), recall (R) and F_1_ score. (F_1_). They are calculated respectively by using the formulas (17)–(19). Precision is the percentage of labels predicted by the model that are correctly predicted. Recall is the number of samples in the sample that are correctly predicted. As precision and recall are mutually exclusive metrics, a combined metric F_1_ score is also needed to judge the recognition performance of the model.

17 $$Precision = \frac{TP}{{TP + FP}}$$

18 $$Recall = \frac{TP}{{TP + FN}}$$

19 $$F_{1} = \frac{2 \times Precision \times Recall}{{Precision + Recall}}$$

where *TP* denotes a positive sample with a correct prediction, *FP* denotes a negative sample with a failed prediction, *FN* denotes a positive sample with a failed prediction and *TN* denotes a negative sample with a correct prediction.

### Dataset

In this paper, we constructed an Aerospace Named Entity Recognition dataset with data from Wikipedia and the China Aerospace News website. It contains 30 k sentences and 53,788 entities. We predefined seven entity types based on the contents of the data, which were labeled by six annotators dividing the work among themselves, and the results were confirmed and validated by a manager. The whole labeling process took about one month. We divide the dataset in the ratio of 8:1:1 to get the training dataset, developing dataset and testing dataset for training and testing our model. The dataset information is shown in Table 1. We used two mainstream Chinese NER datasets, the Weibo dataset^20,21^ and the Resume dataset^1^. The corpus of the Weibo dataset is mainly drawn from social media and contains four types of entities: Person, Location, Organization and Geopolitic. The corpus of the Resume dataset is mainly from Sina Finance. and was made by manually labeling named entities with YEDDA system. Table 2 shows the main information of both datasets.

**Table 2:** Main information of weibo and resume datasets.

Dataset	Type	Train (K)	Dev (K)	Test (K)
Weibo	Sentence	1.4	0.27	0.27
	Char	73.8	14.5	14.8
Resume	Sentence	3.8	0.46	0.48
	Char	124.1	13.9	15.1

### Experimental environment and parameters

In our experiments, we used the same word lexicon and pre-trained character and word embeddings as in the Lattice-LSTM, Radical lexicon from https://github.com/kfcd/chaizi. All comparison model codes are provided by the original authors. Our model was trained on an Ubuntu system using an RTX 3060.

The hyperparameters are set differently for different datasets. The hyperparameter setting for MFT are shown in Table 3. The hyperparameters are set differently for different datasets. On the aerospace dataset MFT consists of 9,765,018 trainable parameters. On the resume dataset, MFT consists of 9,319,506 trainable parameters.

**Table 3:** Hyperparameter setting for MFT.

Dataset	*N*_head_	D_*head*_	D_FFN_	Epoch	Layer	Batch
Aerospace	16	8	384	200	2	5
Weibo	16	8	384	200	1	5
Resume	8	16	384	200	2	10

### Experimental results

In this study, we use the F_1_ score as a criterion for judging the performance of the models, so the precision and recall of the models are the results achieved by the model with the highest F_1_ score on the test set.

#### Aerospace dataset

The experimental results of MFT on the aerospace dataset are shown in Table 4. The experimental results indicate that MFT performs well, with a significant performance improvement of 0.97% in F_1_ score compared to the baseline model FLAT, the recall rate increased by 0.77%, the precision, is 1.16%. LR-CNN and LGN performed worse on the aerospace dataset than on the other datasets, while the LSTM combined with Lattice achieved an F_1_ score of 71.33%, which is 9.88% lower than our MFT model.

**Table 4:** Aerospace NER results.

Models	P	R	F_1_
LatticeLSTM^1^	70.58	72.08	71.33
LR-CNN^12^	70.53	64.35	67.35
LGN^13^	71.66	72.6	72.13
FLAT^3^	80.47	80.01	80.24
MFT	**81.63**	**80.78**	**81.21**
FLAT + BERT^3^	85.33	**85.49**	85.41
MFT + BERT	**86.77**	85.44	**86.10**

Significant values are in bold.

The adoption of the pre-training model BERT by MFT results in a substantial improvement in each performance. Although MFT + BERT does not perform as well as FLAT + BERT in terms of recall, both F_1_ and P have to perform better.

**Figure 8:**
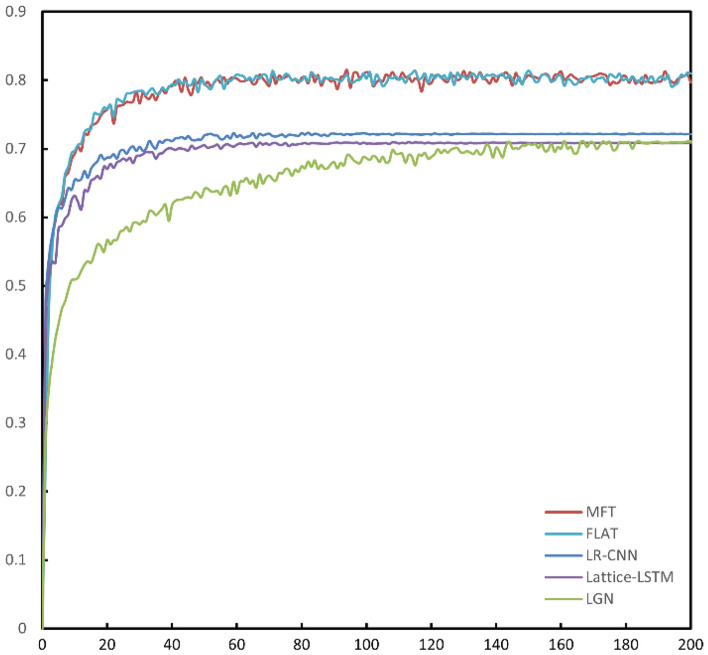
F_1_ Curves during training of all models on the aerospace dataset. MFT's F_1_ curve is essentially above FLAT.

**Figure 9:**
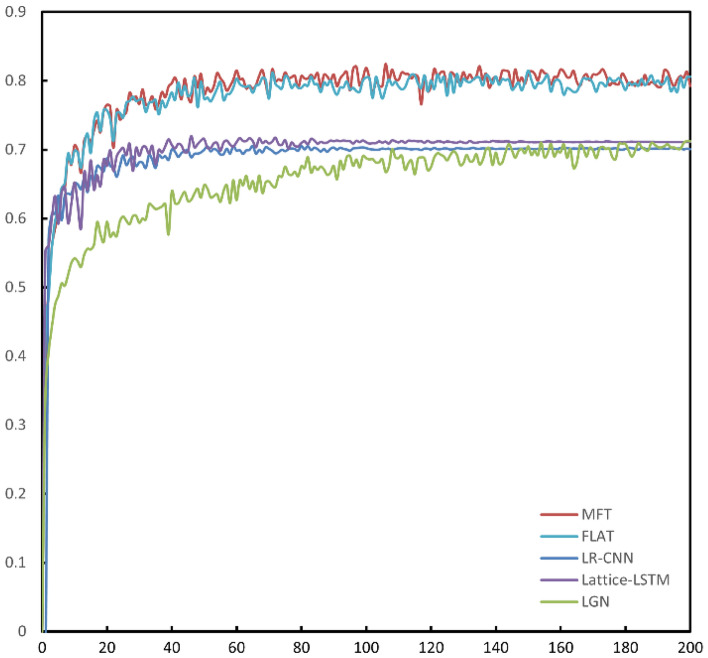
Precision curve during training of the comparison model on the aerospace dataset. MFT has a significantly higher precision rate curve than FLAT.

**Figure 10:**
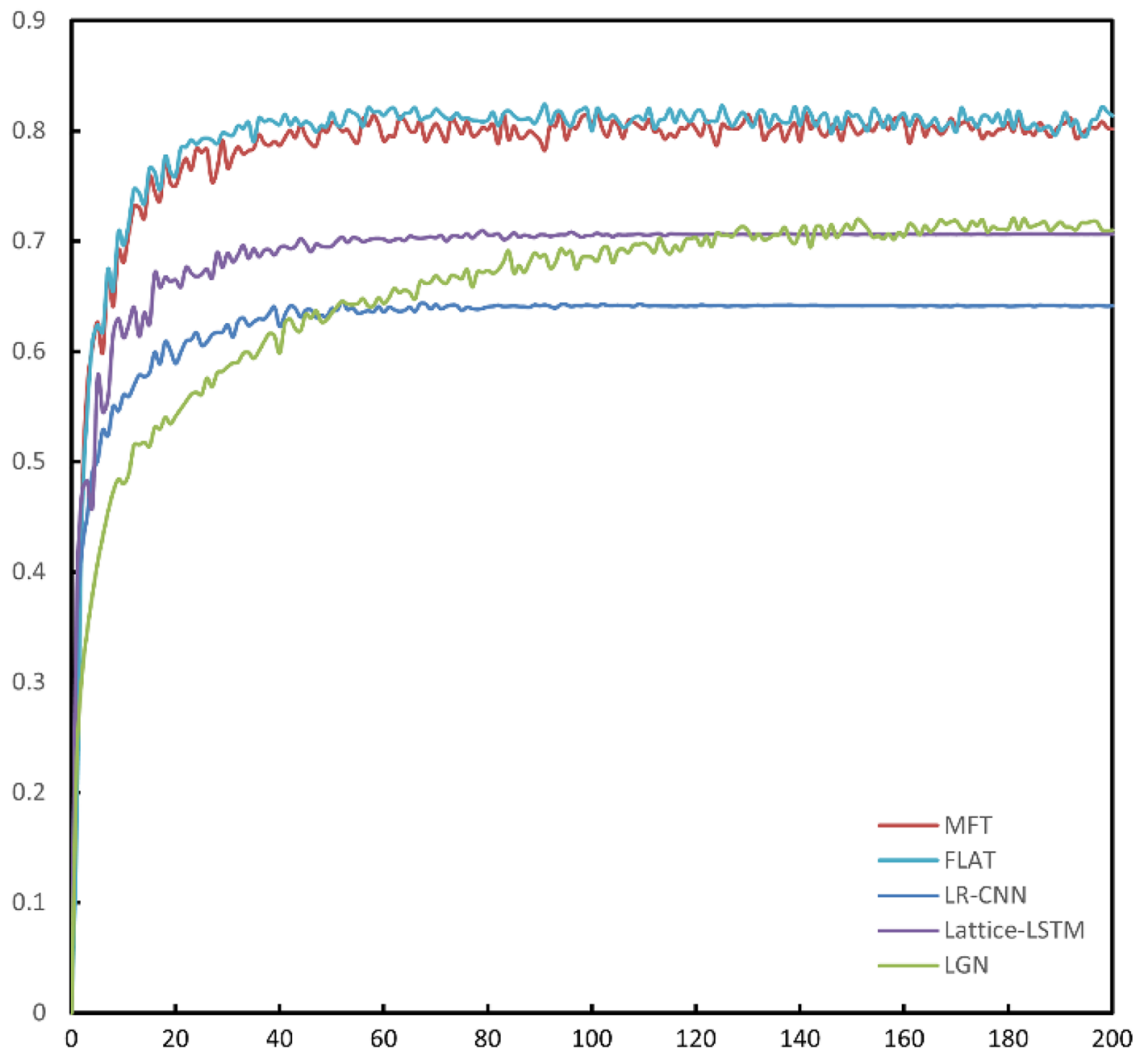
Recall curves during training of all models on the aerospace dataset. MFT's recall is a bit lower than FLAT, but at the 160th epoch it's basically equal to FLAT's level.

Figure 8 shows the F_1_ curve of each model during training on the aerospace dataset, and the performance improvement of MFT in terms of F_1_ score is obvious compared to LGN, Lattice-LSTM and LR-CNN. Compared to FLAT, MFT has a faster improvement in F_1_ score in the early stage of training. From the precision curve of each model in Fig. 9, it can be seen that MFT performs much better than FLAT in terms of precision during the training process, and after the 100th Epoch MFT's precision curve is higher than FLAT's precision curve almost everywhere. However, the recall curves of all models in Fig. 10 show that there is not much difference between the performance of MFT and FLAT with respect to the recall criterion, so the improvement in the overall performance metric F_1_ score of MFT mainly comes from the improvement in the recognition precision of the model.

Table 5 shows the recognition of MFT for different classes of entities on the aerospace dataset. The best recognized entity type is AEAT with F_1_ score of 83.48% followed by TOAV. The worst recognition rate is AASLS with F_1_ score of 61.11% and also AASLS has the least number of entities. Thus the recognition effectiveness of the model is directly related to the amount of data.

**Table 5:** MFT's F_1_ scores for different entity types.

Type	Train	Dev	Test	F_1_
ACAO	2023	254	206	75
AASLS	453	59	54	61.11
TOAV	3467	418	441	80.95
CAS	1413	165	153	64.05
SMAP	7380	897	930	78.49
SAA	550	55	84	61.90
ATAE	25,922	3232	3312	83.48

#### Weibo dataset

Table 6 shows the experimental results of MFT on the Weibo dataset. Compared with other comparison models, MFT has a greater performance improvement with F_1_ score of 64.38%. LR-CNN has the best performance in terms of precision, but the recall rate is 15.03% lower compared to MFT and the F_1_ score is 7.84% lower. The comprehensive performance of the model is improved to a higher level when MFT uses BERT to pre-train the model.

**Table 6:** Weibo NER results.

Models	P	R	F_1_
LatticeLSTM^1^	52.71	53.92	53.13
LR-CNN^12^	**65.06**	50.00	56.54
LGN^13^	**–**	**–**	60.21
FLAT^3^	**–**	**–**	60.32
MFT	63.72	**65.03**	**64.38**
FLAT + BERT^3^	**–**	**–**	68.55
MFT + BERT	**68.33**	**71.09**	**69.71**

Significant values are in bold.

#### Resume dataset

The experimental results of MFT on the Resume dataset are shown in Table 7. The experiments demonstrate that the Double Feed-forward Neural Network and the radical information of Chinese characters do bring performance improvements to the model with F_1_ score of 95.78%, precision of 96.05% and recall rate of 95.52%, all of which are better than other models.

**Table 7:** Main results on resume NER.

Models	P	R	F_1_
LatticeLSTM^1^	94.81	94.11	94.46
LR-CNN^12^	95.37	94.84	95.11
LGN^13^	95.28	95.46	95.37
FLAT^3^	–	–	95.45
MFT	**96.05**	**95.52**	**95.78**
FLAT + BERT^3^	–	–	95.86
MFT + BERT	**96.24**	**95.77**	**96.01**

Significant values are in bold.

### Experiments of feature fusion method

To study the effect on the MFT model after using different fusion methods on *LE* and *RE*, we conducted experiments on MFT on all three datasets. The experimental results are shown in Table 8. On the Weibo dataset and the Resume dataset, concatenating *LE* and *RE* performed better than adding them together. In contrast, For the aerospace dataset, concatenating *LE* and *RE* together still outperforms FLAT despite a decrease in precision, while the F_1_ and recall of MFT are improved, especially the recall by 0.98%.

**Table 8:** Result of different feature fusion method.

Dataset	Method	P	R	F_1_
Aerospace	Concatenation	81.63	80.78	81.21
	Addition	82.03	79.80	80.89
Weibo	Concatenation	63.72	65.03	64.38
	Addition	66.08	58.09	61.83
Resume	Concatenation	96.05	95.52	95.78
	Addition	94.03	93.35	94.72

### Experiments of FFN

The Conformer being used to solve the speech recognition problem contains a double half-step FFN, while the MFT contains a double full-step FFN. In order to verify whether double full-step FFN can bring more performance improvement than double half-step FFN in the named entity recognition task, we set up experiments on the impact of different FFN weight connection methods on the model performance. The experimental results are shown in Table 9. Compared to the double half-step FFN, the double full-step FFN is more suitable for the Named Entity Recognition task.

**Table 9:** Result of different FFN.

Dataset	Type	P	R	F_1_
Aerospace	Double half-step FFN	81.27	79.59	80.43
	Single full-step FFN	82.63	78.85	80.74
	Double full-step FFN	81.63	80.78	81.21
Weibo	Double half-step FFN	66.08	58.09	61.83
	Single full-step FFN	67.14	60.92	63.88
	Double full-step FFN	63.72	65.03	64.38
Resume	Double half-step FFN	94.97	95.09	95.03
	Single full-step FFN	95.64	95.42	95.59
	Double full-step FFN	96.05	95.52	95.78

### Effectiveness study

There are two main improvements of the MFT model, namely, the radical information of Chinese characters was added to enhance the semantics, and double FFN was used to improve the feature encoding capability of the model. In order to verify whether all these improvements bring performance benefits to MFT, we disassemble the model structure and conduct experiments on each of the three datasets. As shown in Table 10, we removed the Double FFN of the MFT and the F_1_ scores of the MFT dropped by 0.47%, 0.5%, 0.19% on the Aerospace, Weibo, and Resume datasets, respectively, after which we proceeded to remove the Radical Feature Module of MFT and revert to FLAT, the F_1_ scores of MFT dropped by 0.5%, 3.56%, 0.14%, respectively. Results in Table show that both improvements on the MFT are effective.

**Table 10:** Result of ablation study.

Dataset	Model architecture	P	R	F_1_
Aerospace	MFT	81.63	80.78	81.21
	Double full-step FFN	82.63	78.85	80.74
	Radical feature module	80.47	80.01	80.24
Weibo	MFT	63.72	65.03	64.38
	Double full-step FFN	67.14	60.92	63.88
	Radical feature module	–	–	60.32
Resume	MFT	96.05	95.52	95.78
	Double full-step FFN	95.64	95.42	95.59
	Radical feature module	–	–	95.45

**Figure 11:**
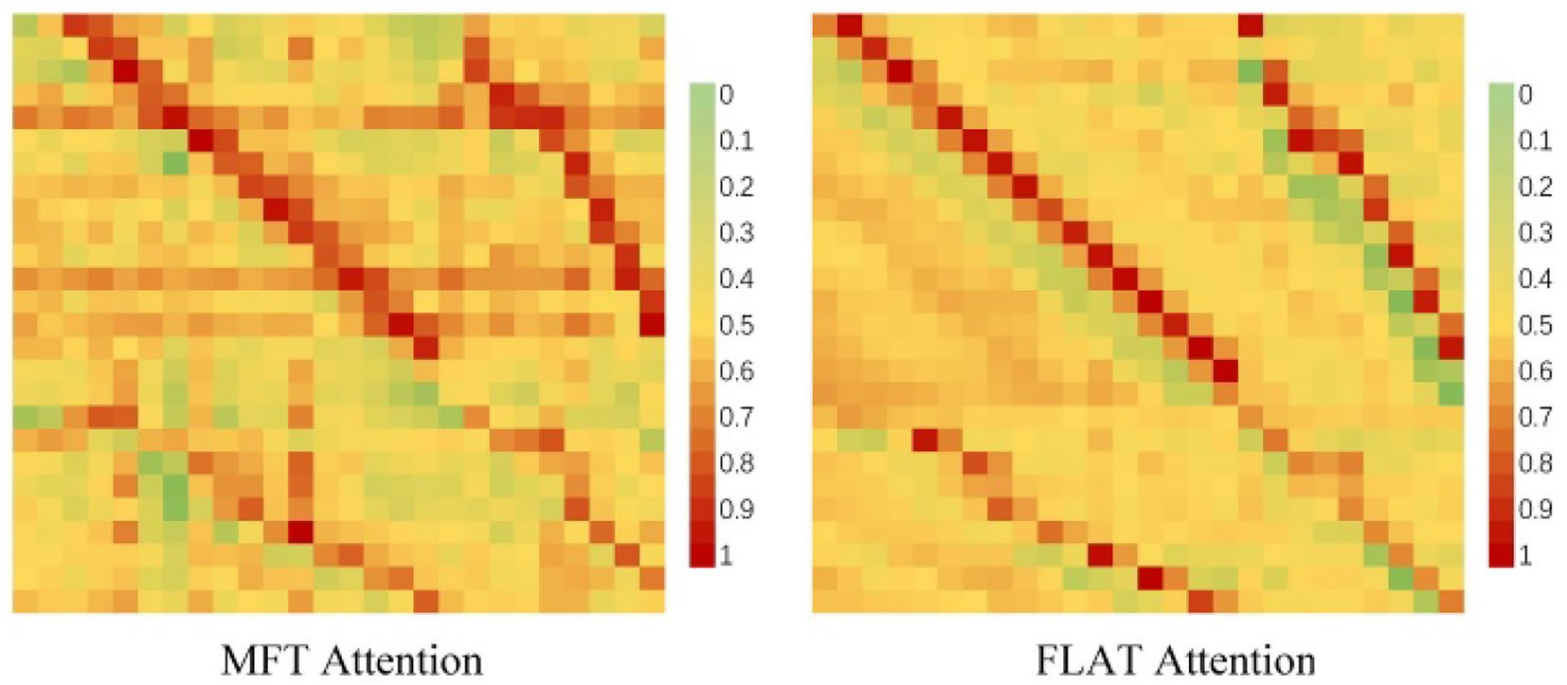
Visualization of attention for MFT and FLAT. MFT has a broader focus and more semantic features are extracted by the self-attentive mechanism.

**Figure 12:**
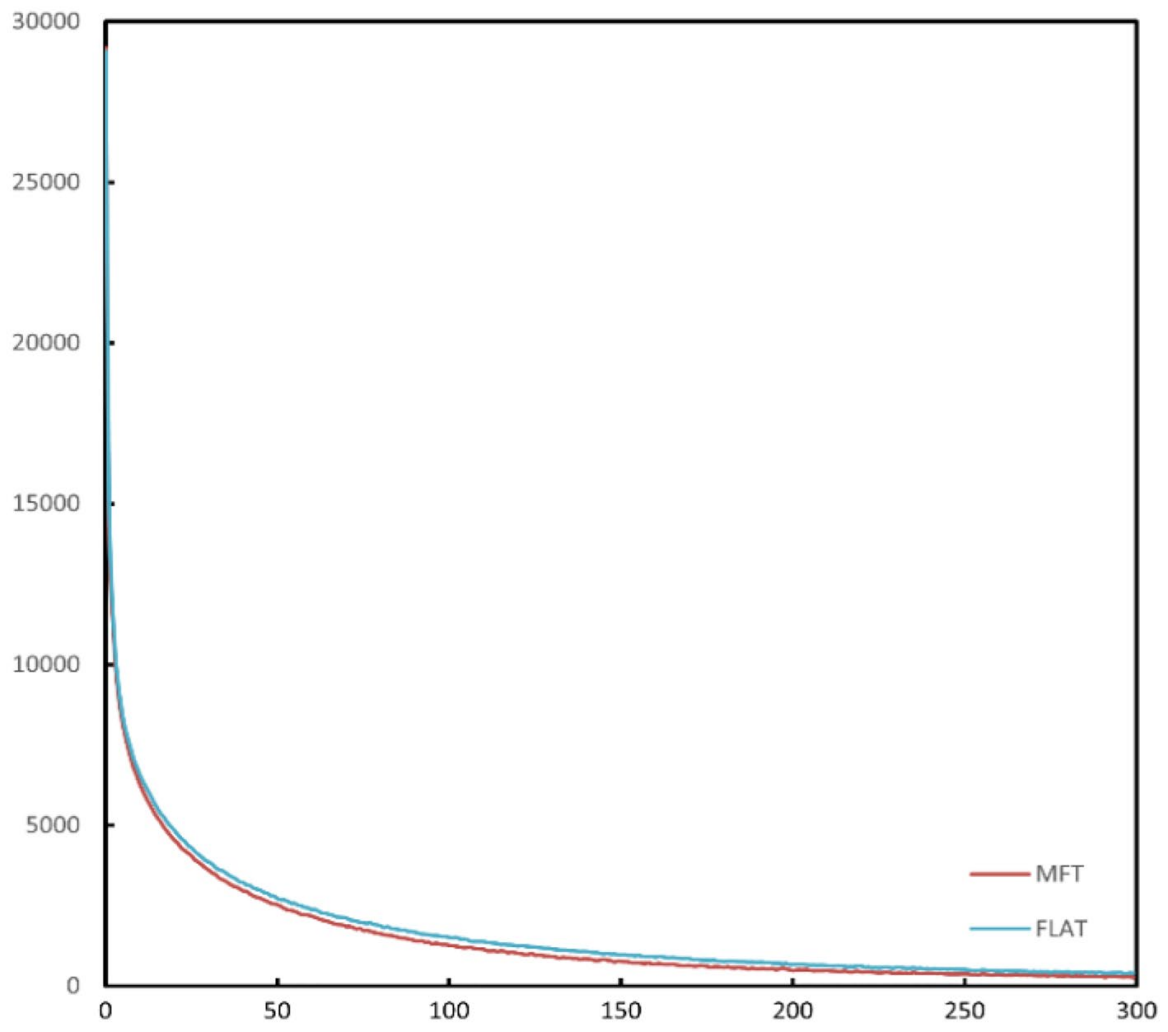
Loss curves for MFT and FLAT. MFT converges faster than the FLAT model and has lower losses.

The effect of the radical feature on the attention of the model is intuitive, as can be seen in Fig. 11, where FLAT has a more focused attention score, while MFT adds extra attention to the information of FLAT. In such a way, the attention to key information is ensured not to be distracted. This allows MFT to converge faster than FLAT during the training of the model, and as shown in Fig. 12, where the loss curve of MFT is lower and decreases faster than that of FLAT.

## Conclusions

In this paper, we propose an Aerospace Named Entity Recognition method based on multi-feature fusion Transformer. Big data from Wikipedia and China Aerospace News are obtained as corpus by crawlers and the aerospace dataset is produced using a manual labelling method. We train and test the MFT on our dataset and the experimental results demonstrate that our model has excellent performance, due to the fact that the radical features of the Chinese characters and the double Feed-forward Neural Network can provide a boost to the recognition rate of the MFT.

In future work, a wider range of Chinese features, such as the pronunciation and graphics of Chinese characters, could also be incorporated for a multimodal approach. However, incorporating more diverse features may introduce invalid elements or noise, which may lead to an increase in model parameters. To mitigate this problem, future work may also require filtering of features to reduce the model size and save computational costs.

## Data Availability

The datasets generated and analyzed during the current study are available in the GitHub repository, https://github.com/Coder-XIAOKAI/Aerospace_NERdatasets.
